# BeadNet: deep learning-based bead detection and counting in low-resolution microscopy images

**DOI:** 10.1093/bioinformatics/btaa594

**Published:** 2020-06-26

**Authors:** Tim Scherr, Karolin Streule, Andreas Bartschat, Moritz Böhland, Johannes Stegmaier, Markus Reischl, Véronique Orian-Rousseau, Ralf Mikut

**Affiliations:** Institute for Automation and Applied Informatics, Eggenstein-Leopoldshafen 76344, Germany; Institute of Biological and Chemical Systems – Functional Molecular Systems, Karlsruhe Institute of Technology, Eggenstein-Leopoldshafen 76344, Germany; Institute for Automation and Applied Informatics, Eggenstein-Leopoldshafen 76344, Germany; Institute for Automation and Applied Informatics, Eggenstein-Leopoldshafen 76344, Germany; Institute of Imaging and Computer Vision, RWTH Aachen University, Aachen 52074, Germany; Institute for Automation and Applied Informatics, Eggenstein-Leopoldshafen 76344, Germany; Institute of Biological and Chemical Systems – Functional Molecular Systems, Karlsruhe Institute of Technology, Eggenstein-Leopoldshafen 76344, Germany; Institute for Automation and Applied Informatics, Eggenstein-Leopoldshafen 76344, Germany

## Abstract

**Motivation:**

An automated counting of beads is required for many high-throughput experiments such as studying mimicked bacterial invasion processes. However, state-of-the-art algorithms under- or overestimate the number of beads in low-resolution images. In addition, expert knowledge is needed to adjust parameters.

**Results:**

In combination with our image labeling tool, BeadNet enables biologists to easily annotate and process their data reducing the expertise required in many existing image analysis pipelines. BeadNet outperforms state-of-the-art-algorithms in terms of missing, added and total amount of beads.

**Availability and implementation:**

BeadNet (software, code and dataset) is available at https://bitbucket.org/t_scherr/beadnet. The image labeling tool is available at https://bitbucket.org/abartschat/imagelabelingtool.

**Supplementary information:**

Supplementary data are available at *Bioinformatics* online.

## 1 Introduction

**Fig. 1.**
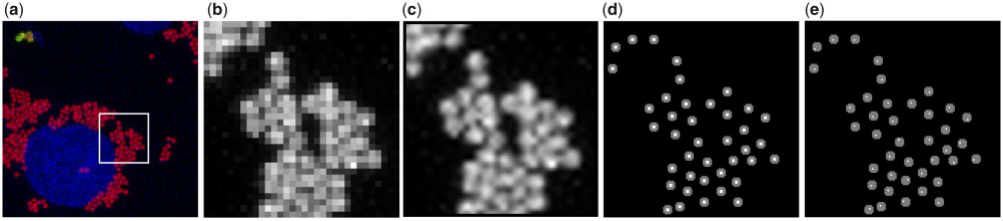
Exemplary application case and detection results of BeadNet. (**a**) A maximum intensity projection of red fluorescent beads (additional green fluorophore for beads outside of cells) and blue fluorescent cell nuclei. The 32 × 32 px test image in (**b**) is taken from the red fluorescent channel of (a). The upsampled test image is shown in (**c**). The white 2 × 2 px seeds in (**d**) are enlarged (gray) for the calculation of the evaluation metrics. The predictions of BeadNet are shown in (**e**) (white in gray ground truth)

Ligand-coupled beads are often used in *in vitro* experiments to mimic bacterial invasion processes (Braun et al., 1998; Hebert et al., 2017; Jung et al., 2009). In the experiments that led to the development of BeadNet, red fluorescent latex beads of 1 µm in size were chemically coupled with a bacterial surface ligand, and their internalization into human cells was investigated. To distinguish internalized beads and beads that remained outside of cells, the cells were fixed without permeabilization, and external beads were recognized using a ligand-specific antibody coupled to a green fluorophore (Fig. 1a).

The complexity of bead detection in low-resolution images may lead biologists to either use an inefficient and user-biased manual quantification or to use a user-friendly but inaccurate method. In addition, the parameters of sophisticated analysis pipelines need to be adjusted in case of changes in the experimental design. Even for experts, it is often easier to annotate new images instead of adjusting parameters for low-resolution and low signal-to-noise-ratio data. Thus, we designed the bead detection and counting software BeadNet to avoid parameter adjustments. For domain adaptation, biologists only need to create new training images using a built-in functionality and annotate them using our labeling tool. Despite its simple use, BeadNet enables a nearly error-free bead detection on a manually annotated low-resolution bead dataset. To our knowledge, this is the first annotated 2D dataset and software tackling the problem of counting beads in low-resolution images with high accuracy. The software combines a crucial upsampling preprocessing with a deep learning-based detection. In addition, BeadNet provides a graphical user interface for easy use, e.g. for generating new training data. The upsampling step and the training data generation are often missing in toolboxes, leaving users alone with the upsampling, the normalization and the creation of new training samples. BeadNet also provides the automatic calculation of evaluation measures and overlays of the detection results.

## 2 Materials and methods

BeadNet consists of an upsampling preprocessing step for low-resolution images and a deep learning-based bead detection with subsequent counting. The bilinear upsampling, hereby, enables distinguishing touching beads (Fig. 1b and c). Furthermore, it eases the annotation of a low-resolution dataset that is required for supervised learning (Fig. 1d). Due to the upsampling preprocessing, centers of touching beads do not share an edge anymore, and the bead detection can be treated as a semantic segmentation task. Thus, for the bead detection, an adapted U-Net trained with a combination of binary cross-entropy and Dice loss is used (Milletari et al., 2016; Ronneberger et al., 2015). Thereby, the traditional upsampling utilizes prior knowledge enforcing the network to work at reasonable scales. To improve the generalization ability of the trained model, specific training data augmentations are applied online during training. To get a more robust seed detection, a morphological dilation with a cross-like mask can be applied to the upsampled training label images. This enlarges the seeds and reduces the number of missing seed detections. However, depending on the resolution and the seeds’ distances the number of missing detections may also increase.

In the post-processing, the raw predictions are binarized using a global threshold of 0.5, and the centroids of predicted beads are calculated. Finally, the detected beads are counted. Additional information concerning the processing steps and the U-Net model used can be found in the Supplementary Information.

To use our BeadNet software, a user needs to select a predefined bead diameter range required for the upsampling preprocessing. If a retraining for domain adaptation is needed, i.e. the resulting seed detection overlays are erroneous, new training data crops can be generated and annotated. Then, multiple models with and without the dilation step are trained and evaluated. For inference, the on the test dataset best performing model is selected automatically.

## 3 Low-resolution bead dataset

We annotated a low-resolution bead dataset consisting of 60 training, 15 validation and 25 test images with 2587 beads in total. The bead diameters range from about 2 px to 4 px (Fig. 1b). Neighboring seeds can touch each other and the center of beads is not well-defined. This makes manual annotation of seeds in the original images very difficult. The application of a fourfold bilinear upsampling and the use of 2 × 2 px seeds enable to annotate beads quickly, to avoid touching seeds and to hit the center easily (Fig. 1c and d).

## 4 Results

For performance measurements, the ground truth seeds are enlarged (Fig. 1d). Then, the normalized amount of missing (no predicted seed in a ground truth bead), split (multiple predicted seeds in a ground truth bead) and added beads (predicted seed in the ground truth background) can be counted. All shown metrics do not count added seeds at the border area (only fully visible beads are annotated, see Supplementary Information for the applied border correction).

Table 1 shows quantitative results of BeadNet, of the Laplacian of Gaussian-based seed detection of TWANG (Bartschat et al., 2016; Stegmaier et al., 2014), of a Hough-transform-based detection using MATLABs imfindcircles (Yuen et al., 1990) and of a simple Otsu thresholding (Otsu, 1979) with Euclidean distance transform on the (upsampled) test dataset. The median BeadNet prediction outperforms the other methods in nearly every metric. More detailed results including the single initializations, and a qualitative comparison with the FISH-quant software for the automatic counting of transcripts in FISH images (Mueller et al., 2013) is provided in the Supplementary Information.

**Table 1.** Results on the 25 test images of the bead dataset (670 beads)

Method	*F*-Score $Q_{F}$	Precision $Q_{P}$	Recall $Q_{R}$	Split $Q_{\text{split}}$ (%)	Missing $Q_{\text{miss}}$ (%)	Added $Q_{\text{add}}$ (%)	Detections (%)
BeadNet	0.971	0.977	0.954	0.45	4.63	1.79	97.76
BeadNet (w. dilation)	0.977	0.979	0.976	0.15	2.39	2.09	99.70
TWANG	0.939	0.960	0.919	0	8.06	3.88	95.82
Hough transform	0.927	0.977	0.882	0	11.79	2.09	90.30
Otsu	0.651	0.766	0.567	2.09	43.28	15.22	74.03

*Note*: For BeadNet, the median of five trained models is shown. The other methods are deterministic.

## 5 Conclusion

BeadNet outperforms traditional bead detection methods, which need expert knowledge to adjust them. The high detection rate shows that no additional preprocessing toolbox is needed, e.g. a 2D adaptation of the image restoration toolbox CSBDeep for the denoising and isotropic recovery of 3D microscopy data (Weigert et al., 2018). Combined with the integrated training data generation enables the high detection rate a user-friendly end-to-end use of BeadNet for the detection of poorly resolved objects, e.g. of ligand-coupled beads or other spherical or non-spherical objects.

## Funding

This work was supported by the Helmholtz Association in the program BioInterfaces in Technology and Medicine (BIFTM), and by a grant from the Deutsche Forschungsgemeinschaft [OR124/16-1 to V.O.-R. and K.S.].

*Conflict of Interest*: none declared.

## Supplementary Material

btaa594_Supplementary_Information
