# Supplementary material for: BeadNet: deep learning-based bead detection and counting in low-resolution microscopy images
Source: Bioinformatics. 2020 Jun 26;36(17):4668–70. doi: 10.1093/bioinformatics/btaa594 (PMC7750944; doi:10.1093/bioinformatics/btaa594)
Supplement: btaa594_Supplementary_Information [file btaa594_supplementary_information.pdf]

BeadNet: Automated bead detection and counting in low-resolution microscopy images

Supplementary information

Tim Scherr[1], Karolin Streule[2], Andreas Bartschat[1], Moritz Böhland[1], Johannes Stegmaier[3], Markus Reischl[1], Véronique Orian-Rousseau[2], and Ralf Mikut[1].

[1] Institute for Automation and Applied Informatics, Karlsruhe Institute of Technology,
[2] Institute of Biological and Chemical Systems – Functional Molecular Systems, Karlsruhe Institute of Technology,
[3] Institute of Imaging and Computer Vision, RWTH Aachen University.

1 Graphical user interface

BeadNet provides a graphical user interface for simple use (Fig. 1). A short but comprehensive step-by-step guide for using BeadNet is available at https://bitbucket.org/t_scherr/beadnet/.

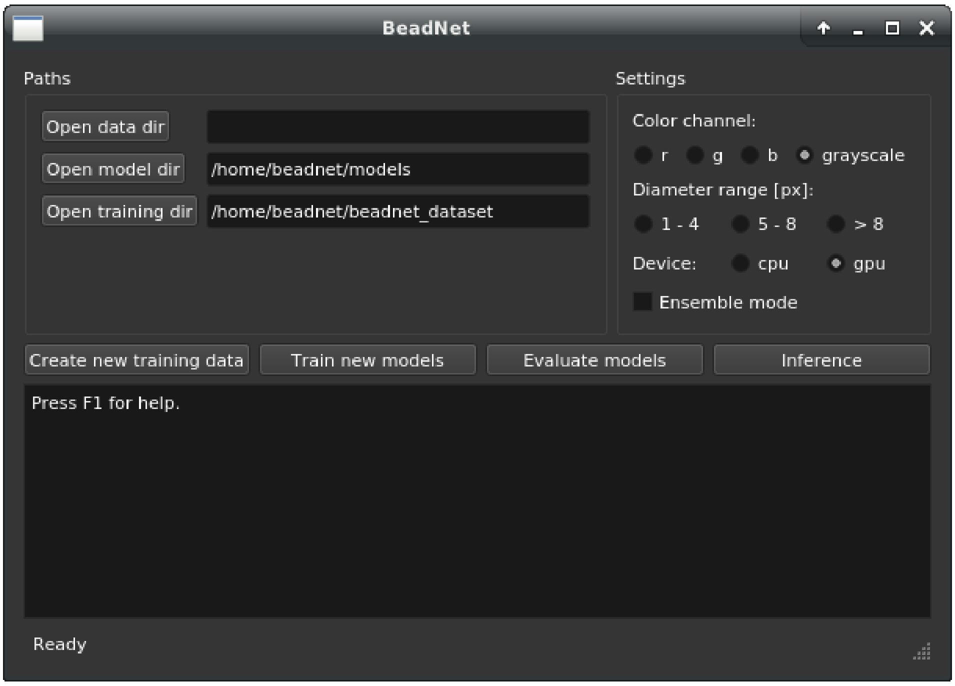

Fig. 1 | Graphical user interface of BeadNet. BeadNet requires no expert knowledge for setting the few settings.

2 Bead data set

The bead data consists of maximum intensity projections of microscopy images showing a mono-layer of ligand-coupled beads marked with a red fluorophore. Due to the trade-offs between measurement speed, image resolution, field of view and data size, the beads in the data set are not resolved well (Fig. 1b in the main paper). For each performed experiment, the maximum intensity projections are min-max normalized independently and converted to uint16 grayscale images.

The normalized maximum intensity projections of five different experiments are used to create 32×32 px crops for training. A sixth experiment is used for the validation set and a seventh for the test set. The crops are upsampled using a bilinear upsampling to 128×128 px images and annotated using our labeling tool (freely available at: https://bitbucket.org/abartschat/imagelabelingtool). Since it is easier to quickly hit the center of the upsampled beads with bigger seeds, 2×2 px seeds were used (Fig. 2). The data set was annotated by two experts who cross-checked their annotations and standardized them together. Table 1 shows the

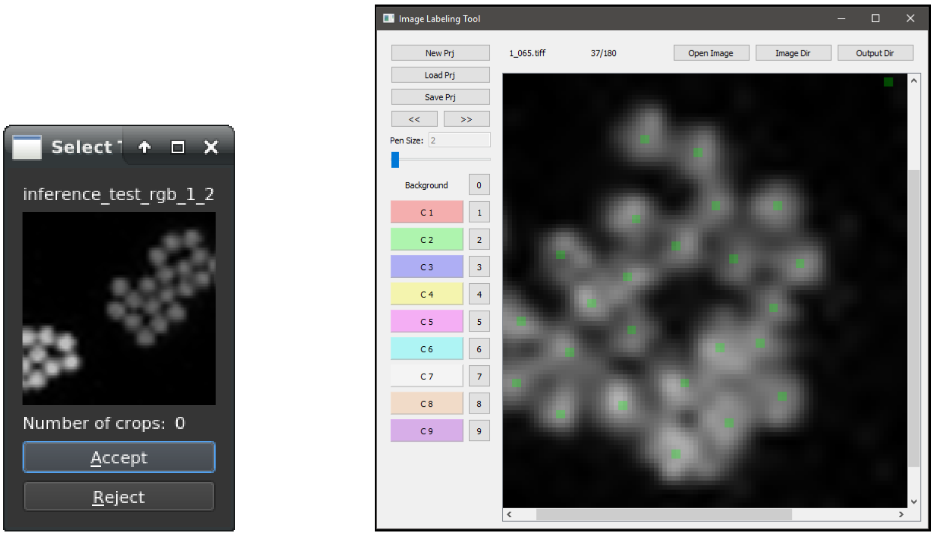

a) BeadNet crop selection

b) Image labeling tool

Fig. 2 | Annotation of new training data. With BeadNet created new training samples (a) can easily be annotated using the image labeling tool (b). See https://bitbucket.org/t_scherr/beadnet/ for more details about this functionality.

Table 1 | Number of annotated beads in the bead data set. $N_{\text{beads}}^{\text{tot}}$ is the total amount of beads in a subset, $N_{\text{beads}}^{\text{min}}$ the minimum amount, $N_{\text{beads}}^{\text{max}}$ the maximum amount and $N_{\text{beads}}^{\text{mean}}$ the mean amount of beads in an image.

Set	Images	Experiments	$N_{\text{beads}}^{\text{tot}}$	$N_{\text{beads}}^{\text{min}}$	$N_{\text{beads}}^{\text{max}}$	$N_{\text{beads}}^{\text{mean}}$
Training	60	1-5	1477	9	57	25
Validation	15	6	440	13	49	29
Test	25	7	670	8	43	27

training, validation and test split of the data set. Additionally, the number of annotated beads in these subsets and the minimum, maximum and mean amount of beads per image are specified.

3 Training process

For the bead detection, BeadNet uses an adapted U-Net convolutional neural network (Ronneberger, Fischer, & Brox, 2015) with batch normalization (Ioffe & Szegedy, 2015) to fix the means and the variances of the layer inputs, transposed convolutions for learnable upsampling and zero-padding in convolutional layers to avoid cropping before concatenating corresponding encoder and decoder feature maps. In the first convolutional layer, 64 feature maps are used. After downsampling using maximum-pooling, the number of feature maps is doubled in the following convolutional layer until 512 feature maps are reached. After a transposed convolution, the number of feature maps is halved in the following convolutional layer. A sigmoid activation function is applied after the output convolutional layer, and ReLU activation function elsewhere.

The network is trained from scratch on the low resolution bead data set using a weighted sum of binary cross-entropy and Dice loss (Milletari, Navab, & Ahmadi, 2016) as loss function, the adaptive Adam optimizer (Kingma & Ba, 2014) in the AMSGrad variant (Reddi, Kale, & Kumar, 2018, learning rate $lr = 6 \cdot 10^{-3}$, $\beta_1 = 0.9$, $\beta_2 = 0.999$, no decay) and a batch size of 4. Dice loss and cross-entropy loss are weighted at a ratio of 2:1. The learning rate is decreased when the validation loss has not decreased for 14 epochs. After 30 epochs without validation loss improvement, the training process is stopped. The model with the best validation loss is used for further analysis.

If the (upsampled) seed distances are large enough, a morphological dilation with a cross-shaped mask can be applied to the label images to get dilated label images (Fig. 3). This can enhance the robustness of the model. The cross-shaped mask prevents diagonal touching seeds.

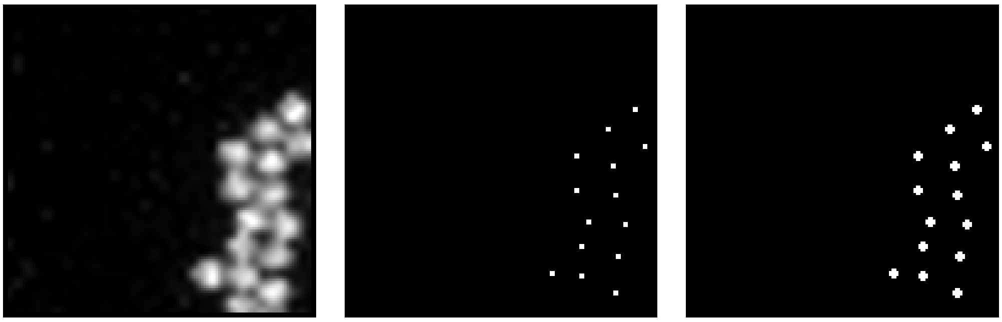

a) Training image b) Label image c) Dilated label image

Fig. 3 | Training data dilation. During training, a morphological dilation with a cross-shaped mask can be applied to the label images (b) to generate the dilated label images (c).

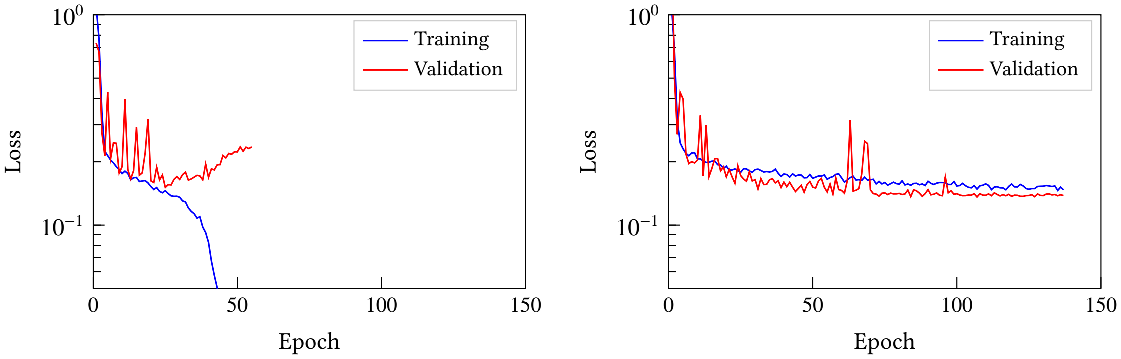

a) Without data augmentation b) With data augmentation

Fig. 4 | Exemplary training and validation loss curves. With data augmentation, the training process is more stable and reliable. Applied are random combinations of flipping, scaling, rotation, contrast, blur and noise augmentations.

To improve the generalization of a trained model to new data, training data augmentation is used. During training, BeadNet applies the following augmentations with probability p to a training image (in this order): flipping ($p = 0.75$), scaling ($p = 0.3$), rotation by a random angle ($p = 0.3$), contrast and gamma adjustments ($p = 0.3$), Gaussian blur ($p = 0.3$) and additive Gaussian noise ($p = 0.3$). BeadNet allows to interactively select and deselect the applied augmentations. Fig. 4 shows that the training process with data augmentation is more stable and reliable than without data augmentation. In addition, overfitting is prevented. Label-preserving augmentations like blur and noise only change the training image and not the label image. Label-changing augmentations require a transform of the label images as well, e.g., rotation and scaling. For the label image transform, nearest neighbor interpolation is used. Fig. 5 shows some exemplary training data augmentations.

4 Compared methods

In this section, the settings of the detection methods which are compared with BeadNet in the main paper are specified. All compared methods are applied to the upsampled images.

4.1 TWANG

TWANG is a segmentation method developed for 2D and 3D cell nuclei segmentation (Stegmaier et al., 2014; Ulman et al., 2017). The Laplacian-of-Gaussian-based seed detection can also be used for bead detection. We used a bead data set adjusted XPIWIT pipeline with median filter pre-processing (Bartschat, Hübner, Reischl, Mikut, & Stegmaier, 2015, $\sigma_{\text{min}} = 2$, $\sigma_{\text{max}} = 3$, $\sigma_{\text{step}} = 1$, NeighborhoodRadius: 3, StdDevMultiplicator: 1, AllowMaximumPlateaus: True, FuseSeedPoints: True).

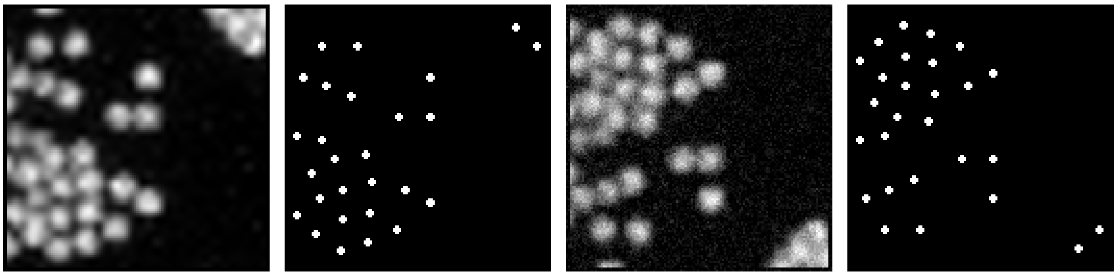

a) Flipping and noise (training image, dilated label image, augmented training image, augmented dilated label image)

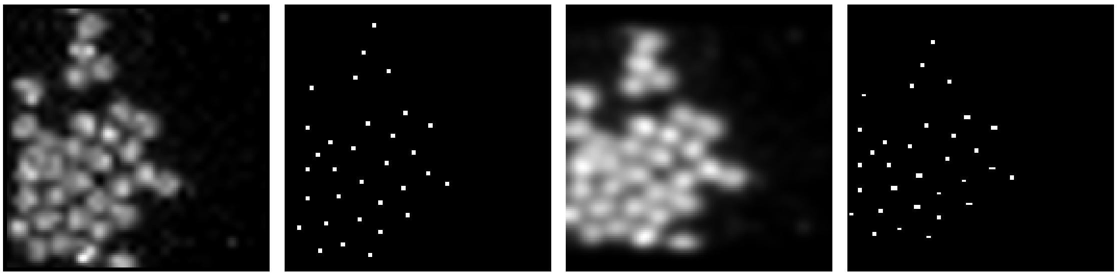

b) Blur, contrast and scaling (training image, label image, augmented training image, augmented label image)

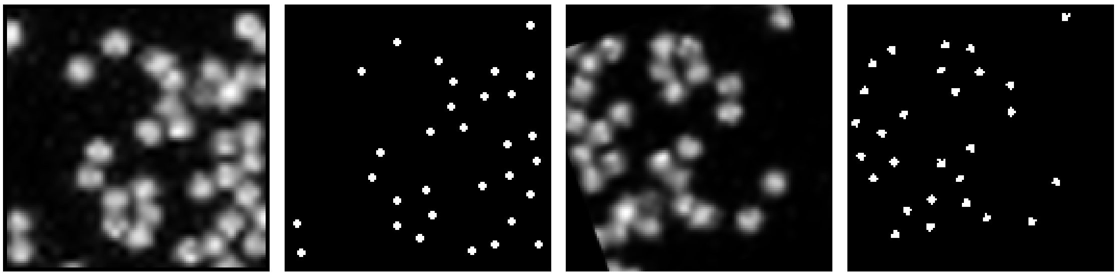

c) Rotation (training image, dilated label image, augmented training image, augmented dilated label image)

Fig. 5 | Training data augmentation. In contrast to label-preserving augmentations (a), label-changing augmentations, e.g., scaling, can change the shape of seeds (b, c). However, we found that both augmentation types improve the training process. Thus, random combinations of flipping, scaling, rotation, contrast, blur and noise augmentations are applied.

4.2 Hough transform

The circular Hough transform can be used to find approximately circular objects in images (Atherton & Kerbyson, 1999; Yuen, Princen, Illingworth, & Kittler, 1990). The accuracy of MATLAB's Hough transform-based spherical object detector `imfindcircles` is limited when the radius of the objects is less than 5 px. Thus, an application to the non-upsampled images is not possible. The parameters of the `imfindcircles` function were manually adjusted to the bead data set (radius range: 5 px - 9 px, sensitivity: 0.9, method: TwoStage, edge threshold: 0.1).

4.3 Otsu

Otsu's method can be used for automatic image thresholding (Otsu, 1979). For the bead detection on the bead data set, the threshold obtained by this method is enlarged with a manually adjusted factor of 1.4. This allows to only binarize the center of the seeds and reduces the merging of beads. After thresholding, an Euclidean distance transform is computed and binarized with an adjusted threshold of 4 to further prevent merging.

5 Metrics

To compare predicted seeds with the ground truth, a radius can be defined to look for a predicted seed. If a predicted seed lies within the circle with the ground truth seed as center, a true positive can be counted.

However, the radius needs to be adjusted to the data set and overlapping circles may occur. Thus, we morphologically dilated label images twice (with a cross-shaped and a square-shaped mask) and used a marker-based watershed with the non-dilated seeds as markers. The resulting ground truth (Fig. 6b) is intensity-coded and no overlapping beads can occur. For many applications, the predicted seeds need to be near the center of the bead. We need the following definitions:

- $N_{\mathrm{gt},i}$: number of ground truth beads in test image i ,
- N_{gt}: number of ground truth beads in the test data set,
- $N_{\mathrm{pred},i}$: number of predicted beads in test image i,
- N_{pred}: number of predicted beads in the test data set,
- $N_{\mathrm{split},i}$: number of split beads (multiple predicted seeds lie in a ground truth bead) in test image i,
- $N_{\mathrm{miss},i}$: number of missing beads (no predicted seed lies in a ground truth bead) in test image i,
- $N_{\mathrm{add},i}$: number of added beads (predicted seed lies in ground truth background) in test image i,
- $tp_i := N_{\mathrm{pred},i} - N_{\mathrm{split},i} - N_{\mathrm{add},i}$: true positives in test image i ,
- $fp_i := N_{\mathrm{split},i} + N_{\mathrm{add},i}$: false positives in test image i,
- $fn_i := N_{\mathrm{miss},i}$: false negatives in test image i.

Merged seeds cannot occur and true negatives are not counted as essentially each undetected background pixel would be a true negative. With that definitions the metrics

$$Q_{\mathrm{split}} = \frac{\sum_i N_{\mathrm{split},i}}{N_{\mathrm{gt}}}, \quad \text{Probability of split beads} \tag{1}$$

$$Q_{\mathrm{miss}} = \frac{\sum_i N_{\mathrm{miss},i}}{N_{\mathrm{gt}}}, \quad \text{Probability of missing beads} \tag{2}$$

$$Q_{\mathrm{add}} = \frac{\sum_i N_{\mathrm{add},i}}{N_{\mathrm{gt}}}, \quad \text{Probability of added beads} \tag{3}$$

$$Q_{\mathrm{P}} = \frac{\sum_i tp_i}{\sum_i tp_i + \sum_i fp_i}, \quad \text{Micro precision} \tag{4}$$

$$Q_{\mathrm{R}} = \frac{\sum_i tp_i}{\sum_i tp_i + \sum_i fn_i}, \quad \text{Micro recall} \tag{5}$$

$$Q_{\mathrm{F}} = \frac{2\, Q_{\mathrm{P}}\, Q_{\mathrm{R}}}{Q_{\mathrm{P}} + Q_{\mathrm{R}}} \quad \text{Micro F-score} \tag{6}$$

can be calculated. Additionally the image-wise averaged metrics

$$\widetilde{Q}_{\mathrm{split}} = \frac{1}{n} \sum_{i=1}^{n} \frac{N_{\mathrm{split},i}}{N_{\mathrm{gt},i}}, \quad \text{Image-wise averaged split beads} \tag{7}$$

$$\widetilde{Q}_{\mathrm{miss}} = \frac{1}{n} \sum_{i=1}^{n} \frac{N_{\mathrm{miss},i}}{N_{\mathrm{gt},i}}, \quad \text{Image-wise averaged missing beads} \tag{8}$$

$$\widetilde{Q}_{\mathrm{add}} = \frac{1}{n} \sum_{i=1}^{n} \frac{N_{\mathrm{add},i}}{N_{\mathrm{gt},i}}, \quad \text{Image-wise averaged added beads} \tag{9}$$

$$\widetilde{Q}_{\mathrm{P}} = \frac{1}{n} \sum_{i=1}^{n} Q_{\mathrm{P},i} = \frac{1}{n} \sum_{i=1}^{n} \frac{tp_i}{tp_i + fp_i}, \quad \text{Macro precision} \tag{10}$$

$$\widetilde{Q}_{\mathrm{R}} = \frac{1}{n} \sum_{i=1}^{n} Q_{\mathrm{R},i} = \frac{1}{n} \sum_{i=1}^{n} \frac{tp_i}{tp_i + fn_i}, \quad \text{Macro recall} \tag{11}$$

$$\widetilde{Q}_{\mathrm{F}} = \frac{1}{n} \sum_{i=1}^{n} \frac{2\, Q_{\mathrm{P},i}\, Q_{\mathrm{R},i}}{Q_{\mathrm{P},i} + Q_{\mathrm{R},i}} \quad \text{Macro F-score} \tag{12}$$

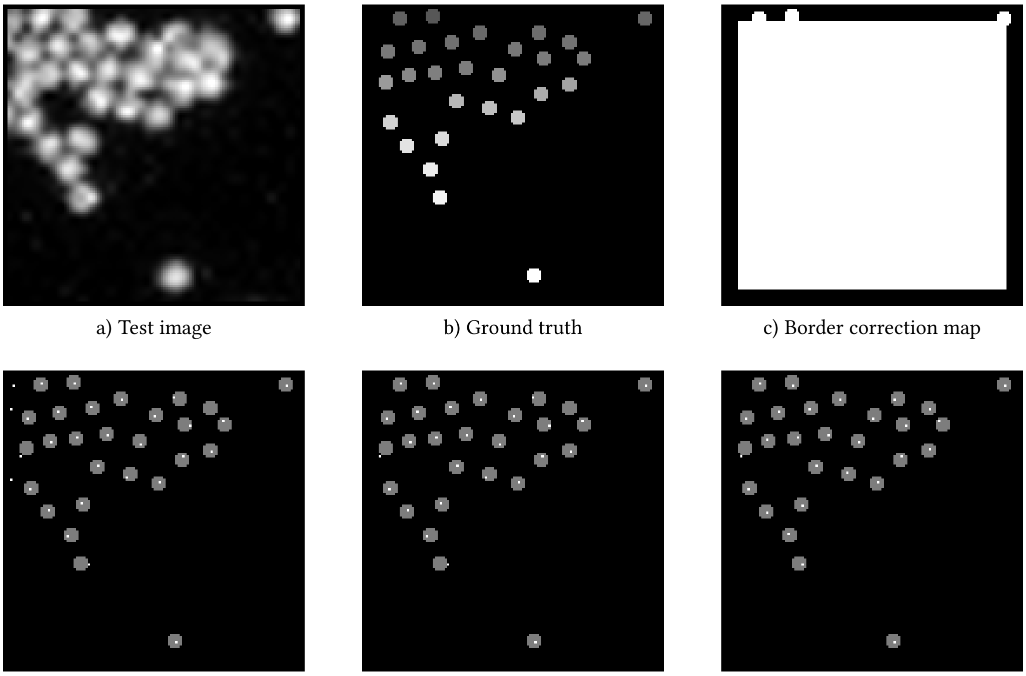

a) Test image b) Ground truth c) Border correction map

d) TWANG e) TWANG border corrected f) BeadNet (dilation)

Fig. 6 | Border correction. Some images also show partially visible beads (a) which are not annotated in the intensity-coded ground truth (b). Using the border correction, only the inner area and annotated border seeds are taken into account (white area in c). After the border correction, predicted border seeds are not considered anymore (d, e). BeadNet is able to learn to predict border seeds or not, depending on the training data and the task (f).

can be calculated. Comparing corresponding metrics gives insight into where errors occur. For example $\widetilde{Q}_{\text{miss}} \gtrless Q_{\text{miss}}$ indicates that missing detections occur mainly on images showing a high/low number of beads.

6 Border correction

In the used low-resolution bead data set, only beads that are at least half on the image are annotated as shown in Fig. 6a-b. Since some detection methods also recognize only partially visible beads, the evaluation metrics are calculated on border-corrected predictions. This enables the comparison of methods. Only the white area shown in Fig. 6c is taken into account (inner area + annotated beads in the border area). Since, the beads have a diameter of about 3 px before and about 12 px after upsampling, we used a 7 px wide border area. In contrast to, e.g., TWANG, BeadNet is capable of learning to detect only partially visible seeds or not depending on the annotated training data (Fig. 6d-f).

7 Results

In addition to Fig. 1 in the main paper, Fig. 7 shows the qualitative bead detection results of the compared methods. TWANG and the Hough transform tend to miss some beads while Otsu's method misses and adds a lot of beads.

Fig. 8, Fig. 9 and Fig. 10 show the evaluation metric scores of BeadNet and the compared methods on the bead data set. BeadNet outperforms the other methods with and without border correction in nearly every metric. Only the probability to split beads is higher. However, the probability to split beads is negligible compared to the probability to miss or add beads and can be decreased using larger seeds. It is also shown that the BeadNet results are almost independent from the initializations. Fig. 11 and Fig. 12 show that there are only small differences between the data set-wise (micro) and image-wise averaged (macro) metrics.

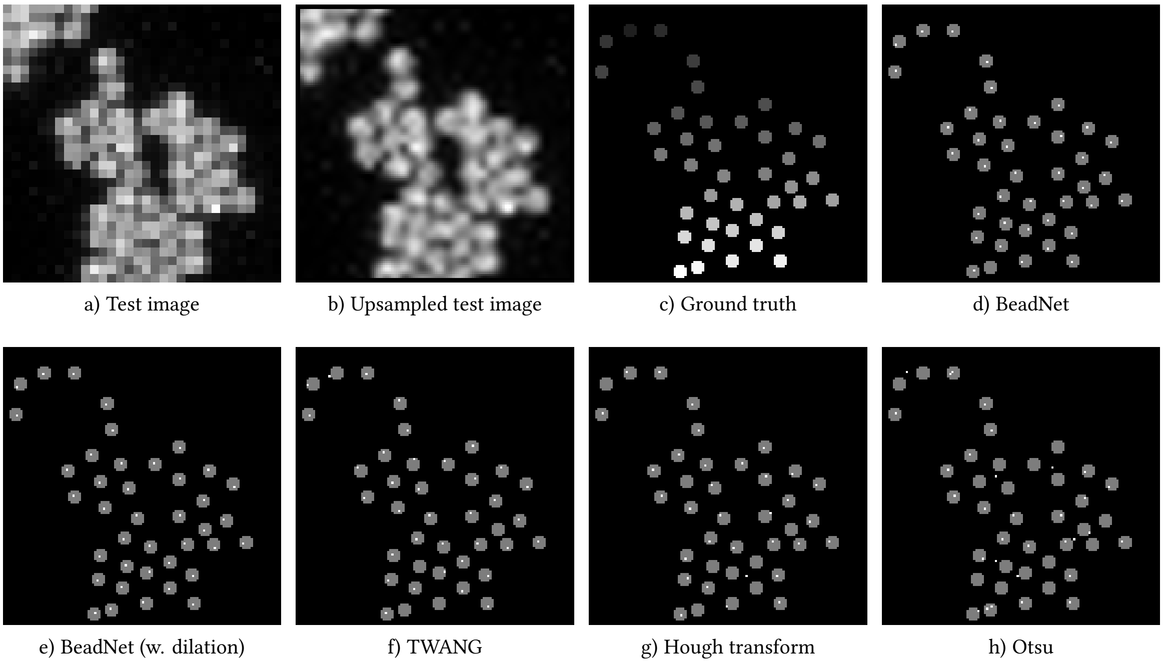

Fig. 7 | Qualitative border corrected bead detection results for various detection methods. The overlay of the predictions (d-h) with the ground truth (c) enables to detect missing, added and split beads easily. BeadNet shows an error-free detection.

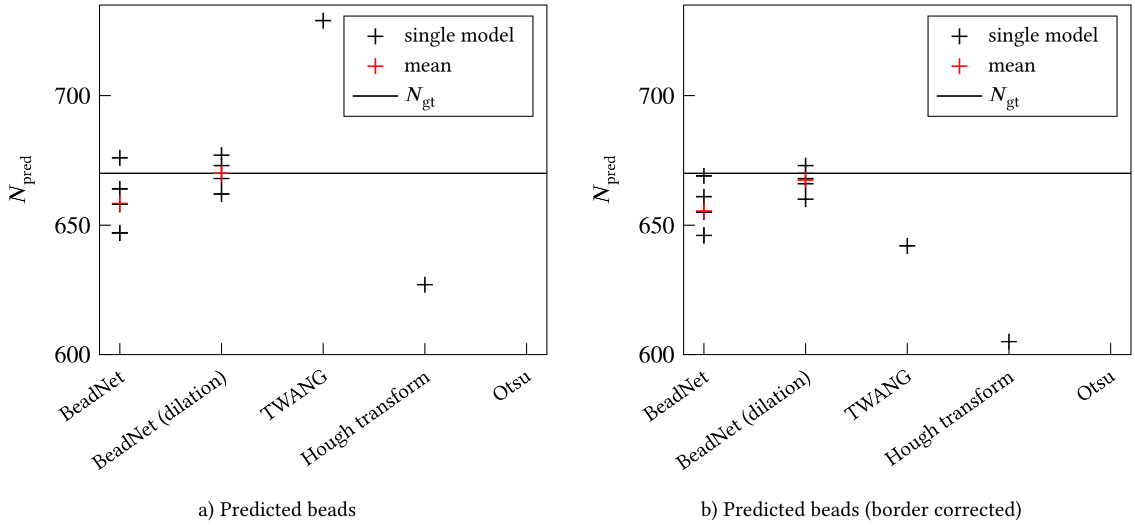

Fig. 8 | Number of predicted beads for various methods on the 25 test images of the bead data set. For BeadNet, the results of five different initializations and the mean are shown. The other methods are deterministic. The number predicted of predicted beads for the Otsu method (497 and 496) are not shown due to visualization reasons.

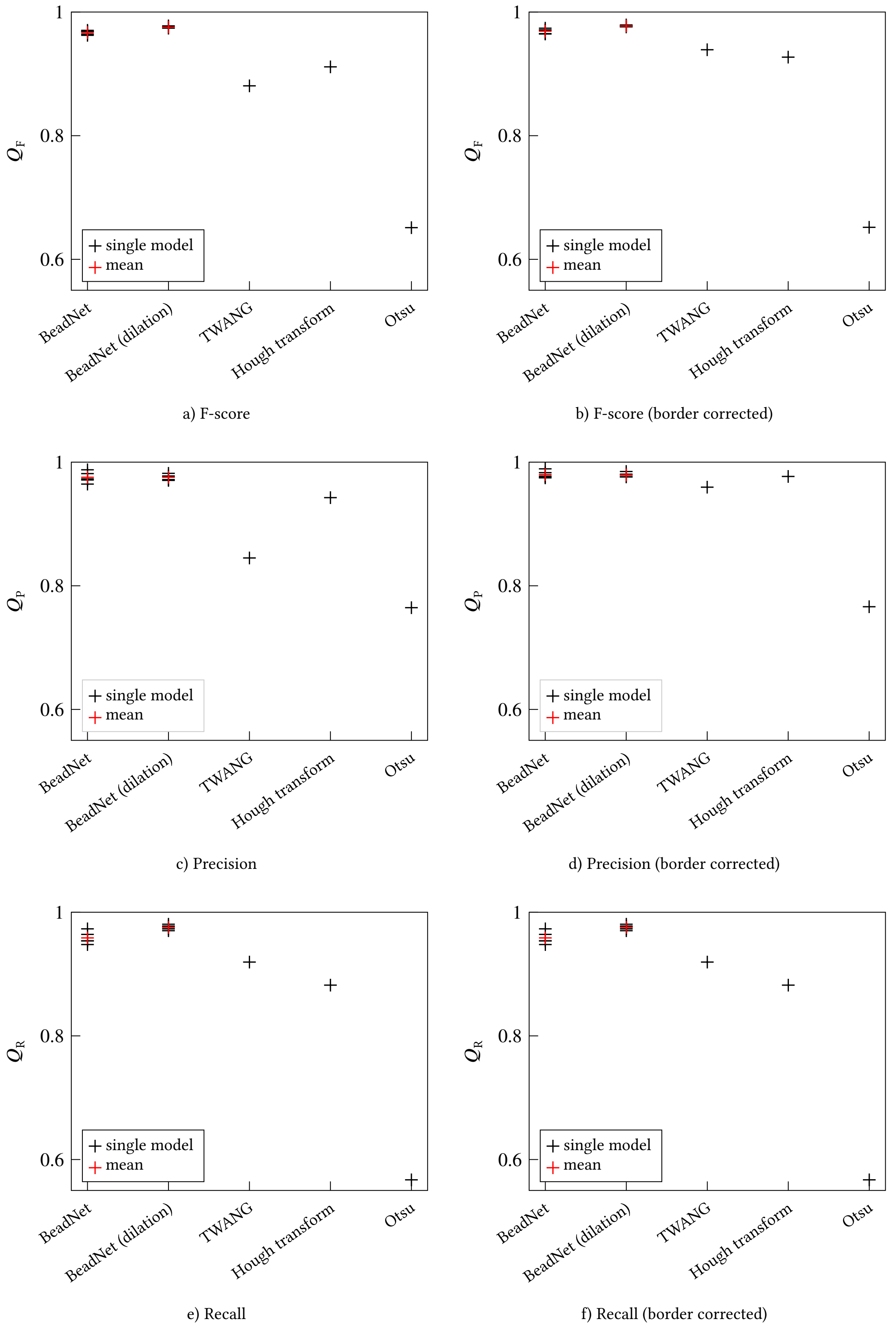

a) F-score
b) F-score (border corrected)
c) Precision
d) Precision (border corrected)
e) Recall
f) Recall (border corrected)

Fig. 9 | Micro precision, recall and F-score of various methods on the 25 test images of the bead data set. For BeadNet, the results of five different initializations and the mean are shown. The other methods are deterministic.

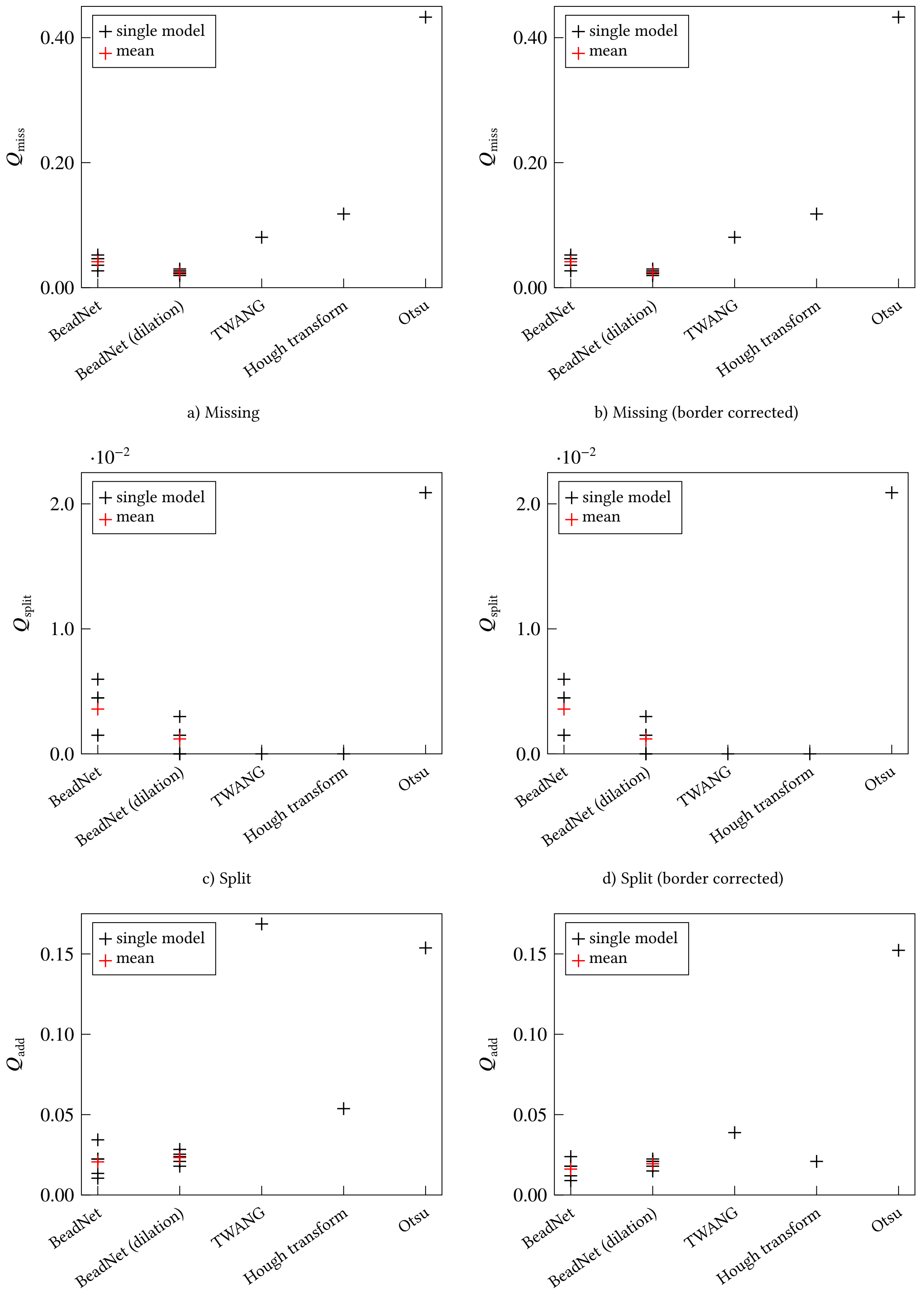

a) Missing

b) Missing (border corrected)

c) Split

d) Split (border corrected)

e) Added

f) Added (border corrected)

Fig. 10 | Split, added and missing beads of various methods on the 25 test images of the bead data set. For BeadNet, the results of five different initializations and the mean are shown. The other methods are deterministic.

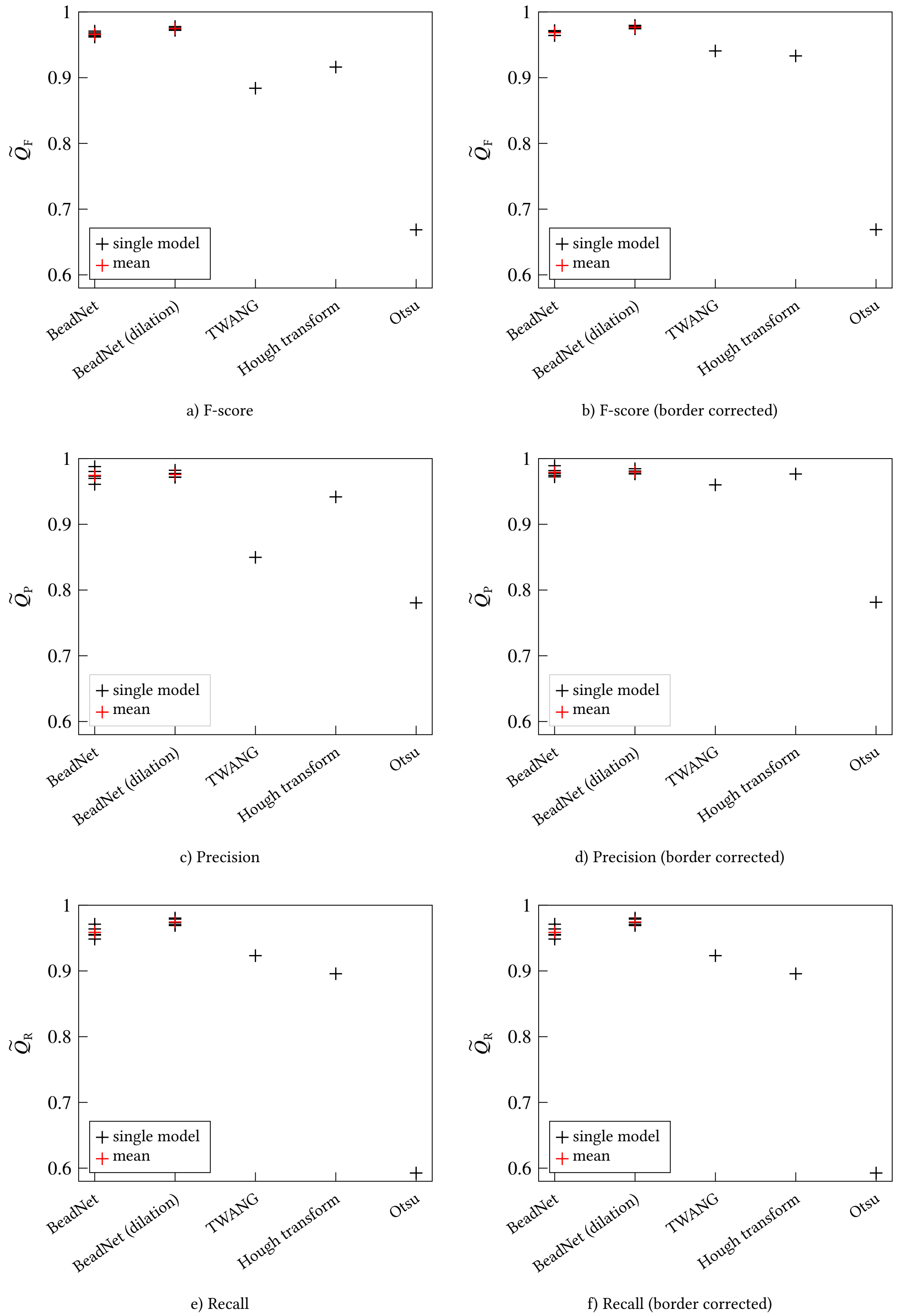

Fig. 11 | Macro precision, recall and F-score of various methods on the 25 test images of the bead data set. For BeadNet, the results of five different initializations and the mean are shown. The other methods are deterministic.

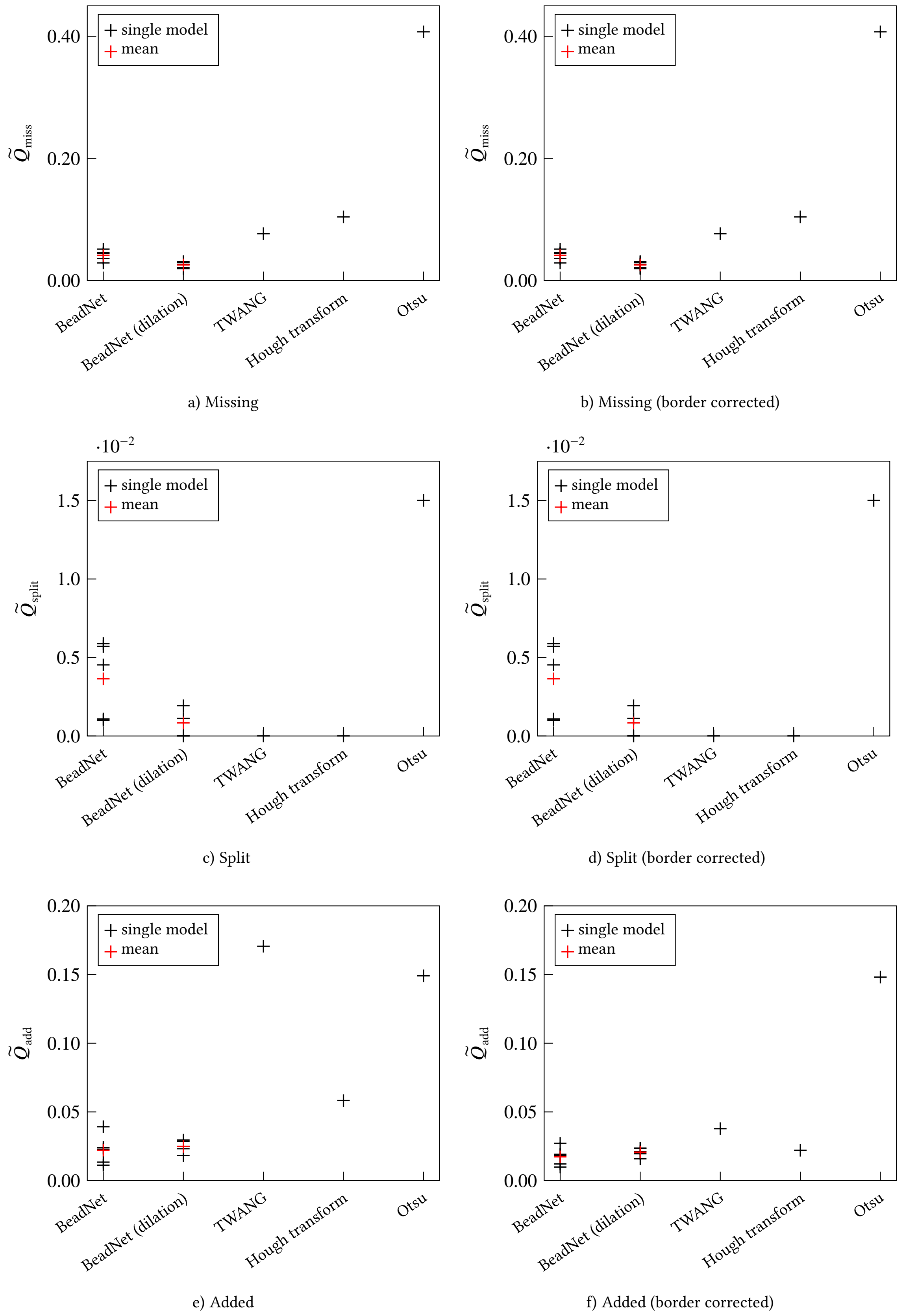

Fig. 12 | Split, added and missing beads of various methods on the 25 test images of the bead data set (image-wise). For BeadNet, the results of five different initializations and the mean are shown. The other methods are deterministic.

8 Qualitative Comparison with FISH-quant

FISH-quant[1] is a tool for the automatic counting of transcripts in 3D FISH (fluorescence in situ hybridization) images (Mueller et al., 2013). For spot detection, 3D Gaussians are fitted to fluorescent spots. Fig. 13 shows an example image and Fig. 14 shows the FISH-quant result provided with the software. Since the software groups the detection to manually drawn or automatically detected cells, the result for only one cell is shown.

To apply BeadNet to the 3D FISH-quant example images, we created maximum intensity projections using Fiji (https://imagej.net/Fiji). A few very bright spots do not allow an adequate normalization of the spots to detect. Therefore, we clipped the intensity values using Fiji enabling a better normalization, visualization and annotation. The provided pre-trained BeadNet models tend to overestimate the number of spots on the FISH-quant images since spots and background differ much from the bead training data. Thus, we generated 20 new training crops (diameter range: 1px - 4px), annotated them, and added them to the bead data set. Using the graphical user interface of BeadNet, this requires neither expert knowledge nor much time. Then, new models were trained and evaluated using standard BeadNet settings. Fig. 15 shows the BeadNet results on a FISH-quant example image that has not been used for the training data generation. The BeadNet results are competitive to the FISH-quant results but less expert knowledge is required for parameter adjustments. Since no manually from experts annotated ground truth data are available, a quantitative comparison in terms of missing, split and added object is not possible.

References

Atherton, T., & Kerbyson, D. (1999). Size Invariant Circle Detection. *Image and Vision Computing*, *17*, 795–803. doi:10.1016/S0262-8856(98)00160-7

Bartschat, A., Hübner, E., Reischl, M., Mikut, R., & Stegmaier, J. (2015). XPIWIT—an XML Pipeline Wrapper for the Insight Toolkit. *Bioinformatics*, *32*, 315–317. doi:10.1093/bioinformatics/btv559

Ioffe, S., & Szegedy, C. (2015). Batch Normalization: Accelerating Deep Network Training by Reducing Internal Covariate Shift. In *Proceedings of the 32nd International Conference on Machine Learning* (Vol. 37, pp. 448–456). PMLR. arXiv: 1502.03167v3

Kingma, D., & Ba, J. (2014). Adam: A Method for Stochastic Optimization. arXiv: 1412.6980v9

Milletari, F., Navab, N., & Ahmadi, S. A. (2016). V-Net: Fully Convolutional Neural Networks for Volumetric Medical Image Segmentation. In *2016 Fourth International Conference on 3D Vision* (pp. 565–571). doi:10.1109/3DV.2016.79

Mueller, F., Senecal, A., Tantale, K., Marie-Nelly, H., Ly, N., Collin, O., … Zimmer, C. (2013). FISH-quant: Automatic Counting of Transcripts in 3D FISH Images. *Nat. Methods*, *10*, 277–278. doi:10.1038/nmeth.2406

Otsu, N. (1979). A Threshold Selection Method from Gray-Level Histograms. *IEEE Transactions on Systems, Man, and Cybernetics*, *9*, 62–66. doi:10.1109/TSMC.1979.4310076

Reddi, S. J., Kale, S., & Kumar, S. (2018). On the Convergence of Adam and Beyond. In *International Conference on Learning Representations*. arXiv: 1904.09237v1

Ronneberger, O., Fischer, P., & Brox, T. (2015). U-Net: Convolutional Networks for Biomedical Image Segmentation. In *Medical Image Computing and Computer-Assisted Intervention – MICCAI 2015* (pp. 234–241). doi:10.1007/978-3-319-24574-4_28

Stegmaier, J., Otte, J. C., Kobitski, A., Bartschat, A., Garcia, A., Nienhaus, G. U., … Mikut, R. (2014). Fast Segmentation of Stained Nuclei in Terabyte-scale, Time Resolved 3D Microscopy Image Stacks. *PLOS ONE*, *9*, 1–11. doi:10.1371/journal.pone.0090036

Ulman, V., Maška, M., Magnusson, K. E., Ronneberger, O., Haubold, C., Harder, N., … Radojevic, M., et al. (2017). An Objective Comparison of Cell-Tracking Algorithms. *Nature Methods*, *14*, 1141–1152. doi:10.1038/nmeth.4473

Yuen, H. K., Princen, J., Illingworth, J., & Kittler, J. (1990). Comparative Study of Hough Transform Methods for Circle Finding. *Image and Vision Computing*, *8*, 71–77. doi:10.1016/0262-8856(90)90059-E

[1] https://bitbucket.org/muellerflorian/fish_quant/

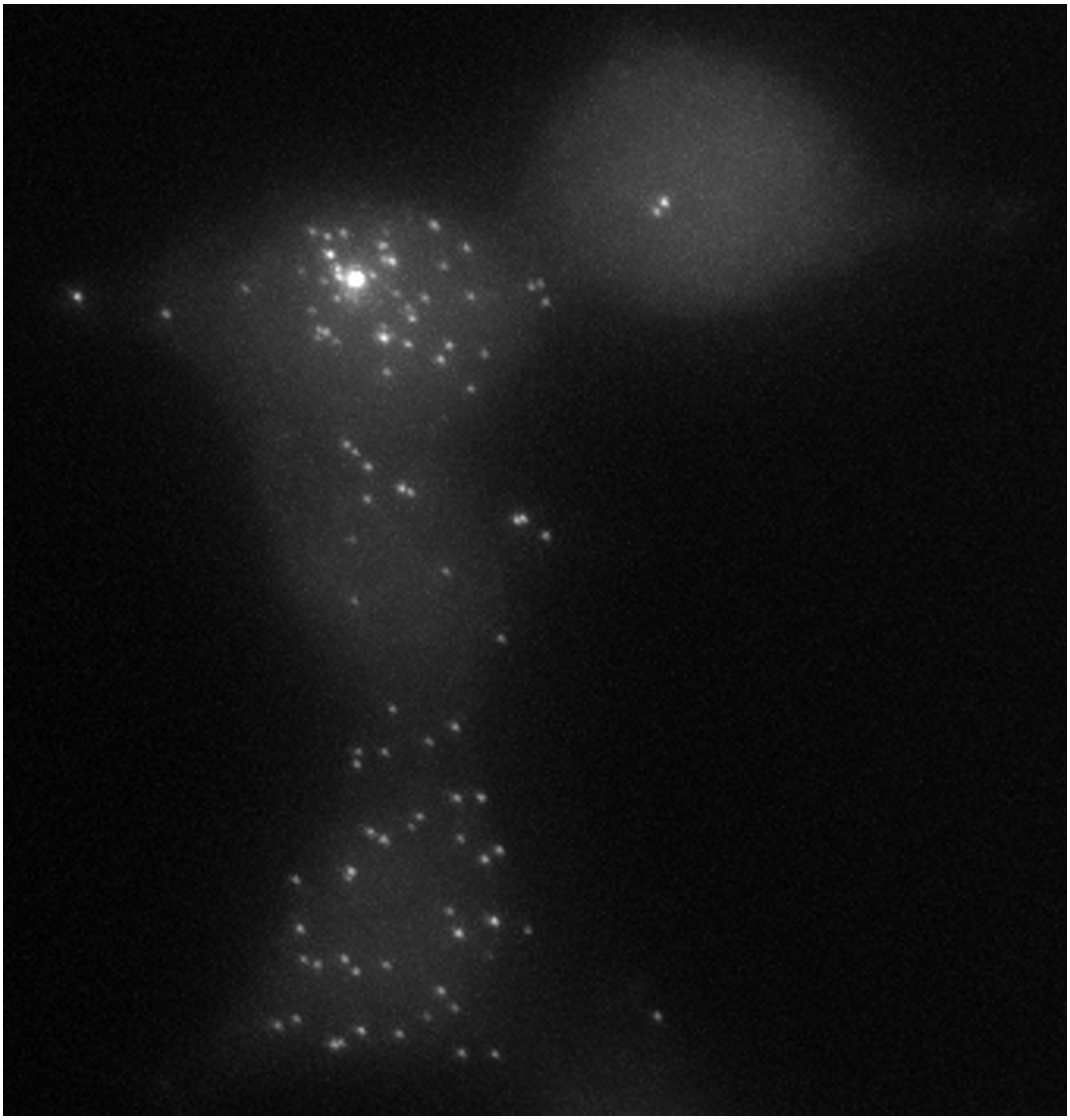

Fig. 13 | FISH-quant test image.

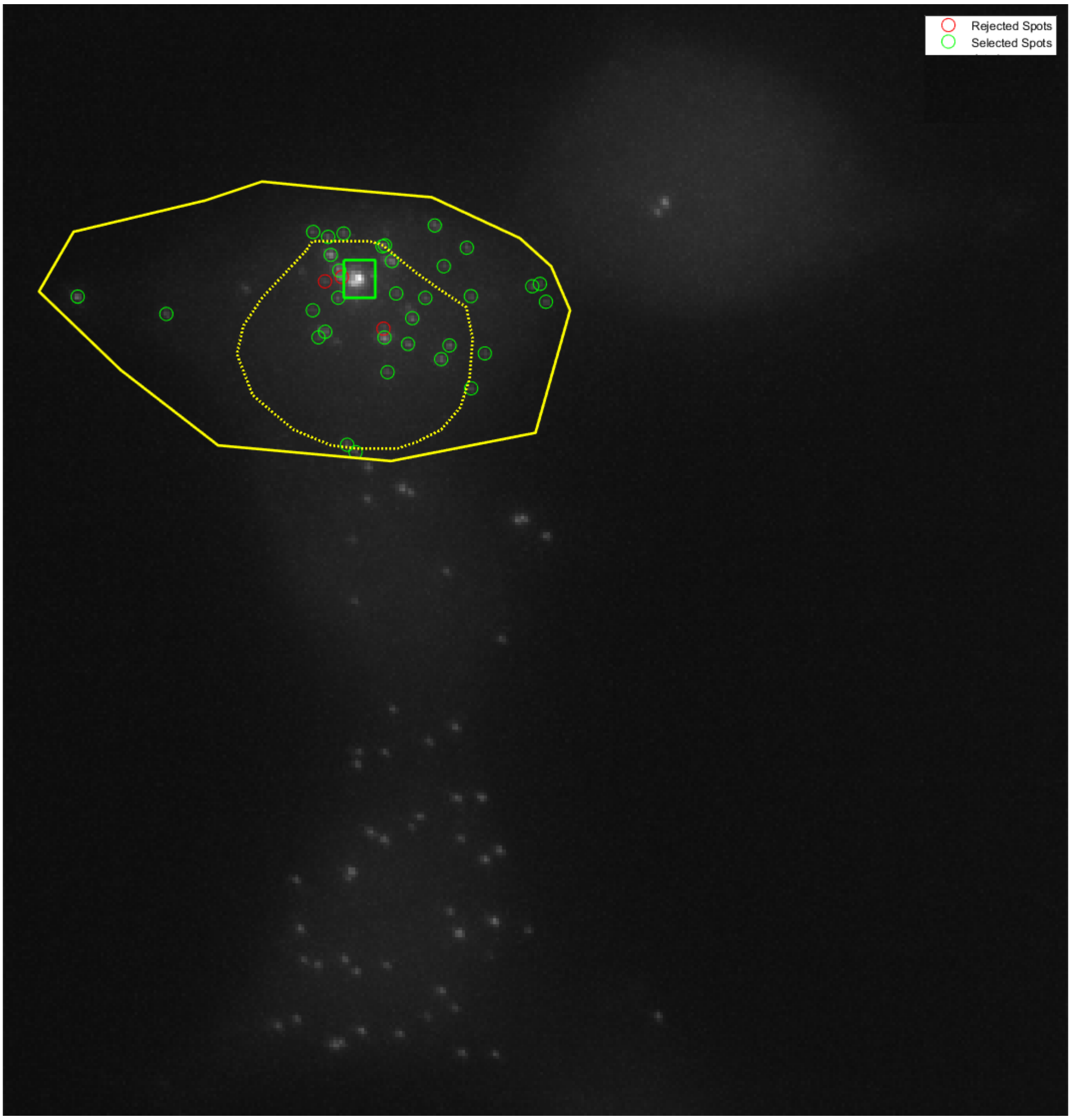

Fig. 14 | FISH-quant spot detection. The FISH-quant software uses an adjustable quality score to select and reject spots. Only detected spots within the yellow drawn cell are shown.

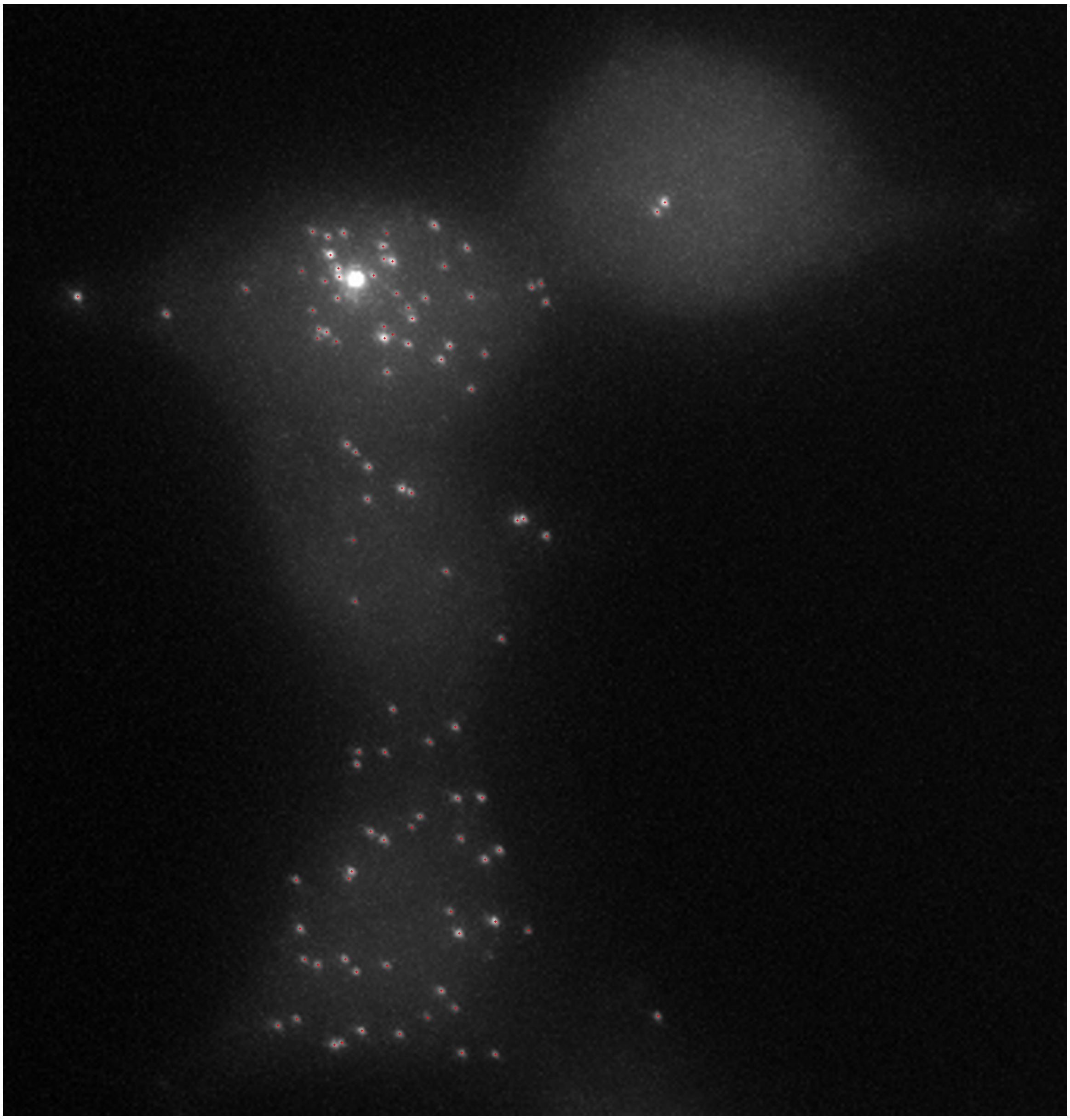

Fig. 15 | BeadNet spot detection on the FISH-quant test image. BeadNet is also capable to predict some spots not detected by FISH-quant in Fig. 14.
